# A Screen for PKN3 Substrates Reveals an Activating Phosphorylation of ARHGAP18

**DOI:** 10.3390/ijms21207769

**Published:** 2020-10-20

**Authors:** Michal Dibus, Jan Brábek, Daniel Rösel

**Affiliations:** 1 Department of Cell Biology, Charles University, Viničná 7, 12800 Prague, Czech Republic; michal.dibus@natur.cuni.cz (M.D.); brabek@natur.cuni.cz (J.B.); 2 Biotechnology and Biomedicine Centre of the Academy of Sciences and Charles University (BIOCEV), Průmyslová 595, 25242 Vestec u Prahy, Czech Republic

**Keywords:** PKN3, phosphorylation, phosphoproteomic screen, ARHGAP18, Rho-GTP

## Abstract

Protein kinase N3 (PKN3) is a serine/threonine kinase implicated in tumor progression of multiple cancer types, however, its substrates and effector proteins still remain largely understudied. In the present work we aimed to identify novel PKN3 substrates in a phosphoproteomic screen using analog sensitive PKN3. Among the identified putative substrates we selected ARHGAP18, a protein from RhoGAP family, for validation of the screen and further study. We confirmed that PKN3 can phosphorylate ARHGAP18 in vitro and we also characterized the interaction of the two proteins, which is mediated via the *N*-terminal part of ARHGAP18. We present strong evidence that PKN3-ARHGAP18 interaction is increased upon ARHGAP18 phosphorylation and that the phosphorylation of ARHGAP18 by PKN3 enhances its GAP domain activity and contributes to negative regulation of active RhoA. Taken together, we identified new set of potential PKN3 substrates and revealed a new negative feedback regulatory mechanism of Rho signaling mediated by PKN3-induced ARHGAP18 activation.

## 1. Introduction

PKN3 (protein kinase N3) is a serine/threonine kinase belonging to the PKN family of kinases that act downstream of small Rho GTPases. While the expression of PKN1 and PKN2 is ubiquitous in most of the adult tissues [1,2,3], the expression of PKN3 is restricted mainly to endothelial cells [4], osteoclasts [5,6] and several cancer cell types [7,8,9]. In endothelial cells, downregulation of PKN3 was shown to block cell migration and formation of tubular structures in both 2D and 3D as a result of impaired actin reorganization [4,10]. Selective targeting of PKN3 expression in endothelial cells by systemic administration of small liposomal siRNA, Atu027, impaired the formation of micro- and macro-metastases in lungs in both experimental and spontaneous metastasis mouse models [11]. In cancer cells, PKN3 was shown to act downstream of activated PI3K (phosphatidylinositol 3-kinase) promoting the malignant progression of prostate cancer [8]. Moreover, downregulation of PKN3 expression led to impaired primary tumor growth and inhibition of metastasis in breast and prostate cancer [9]. Recently, we have identified interaction between PKN3 and adaptor protein p130Cas (Crk-associated substrate; BCAR1 in human) [12] which promotes the pro-malignant growth of cancer cells and could partly explain PKN3-mediated phenotype [13]. The effect of PKN3 expression on regulation of cancer development was further highlighted in PKN3 knock-out mice that showed slower rates of leukemia development induced by the loss of PTEN (phosphatase and tensin homolog) [14] and a decrease in the number of secondary tumor sites [15].

Since PKN3 downstream signaling still remains largely understudied, we decided to perform a phosphoproteomic screen to identify new PKN3 substrates using a chemical genetic approach based on the mutation in gatekeeper residue of the kinase [16,17]. Among the newly identified putative substrates of PKN3, we selected ARHGAP18 (Rho GTPase Activating Protein 18) for validation of the screen and further study. 

ARHGAP18—also known as SENEX—is a member of a RhoGAP protein family which plays a role in regulation of activity of small Rho GTPases. Interestingly, ARHGAP18 exhibits different specificity towards individual Rho GTPases. In endothelial cells, ARHGAP18 was shown to act preferentially on RhoC [18], whereas in cancer cells it shows specificity mainly for RhoA [19]. However, recent findings suggest RhoA activity could also be regulated by ARHGAP18 in endothelial cells [20]. ARHGAP18 was found to regulate cell polarization, cell shape and migration of cancer cells [19]. Moreover, it was shown to act downstream of YAP (Yes-associated protein) in regulation of cell shape and tissue tension homeostasis in development [21] and to regulate actin cytoskeleton organization downstream of IP3R3 (inositol 1,4,5-trisphosphate receptor 3) via the RhoA/mDia1/FAK signaling pathway [22]. It associates with microtubules in a GAP domain-dependent manner [23] and localizes to the leading edge during cell spreading and migration [19]. However, the effect of ARHGAP18 expression in cancer cells is still contradictory in individual studies, since inhibition of cancer cell migration, invasion and tumor growth was observed after both overexpression [24] and downregulation [25,26] of ARHGAP18. Moreover, high levels of ARHGAP18 expression were associated with better outcome [27], as well as with worse metastasis-free and overall survival [25]. In endothelial cells, expression of ARHGAP18 contributes to vascular stabilization and acts as a negative regulator of angiogenesis [18]. Overexpression of ARHGAP18 leads to premature senescence of endothelial cells [28]. Recently, it was shown to facilitate athero-protective endothelial cell alignment in response to laminar shear flow [29,30]. 

In our study, we found that PKN3 interacts with ARHGAP18 and we present strong evidence that PKN3 phosphorylation of ARHGAP18 leads to the activation of its GAP domain, resulting in a decrease of RhoA activity.

## 2. Results

### 2.1. Phosphoproteomic Screen for Novel PKN3 Substrates

**Figure 1:**
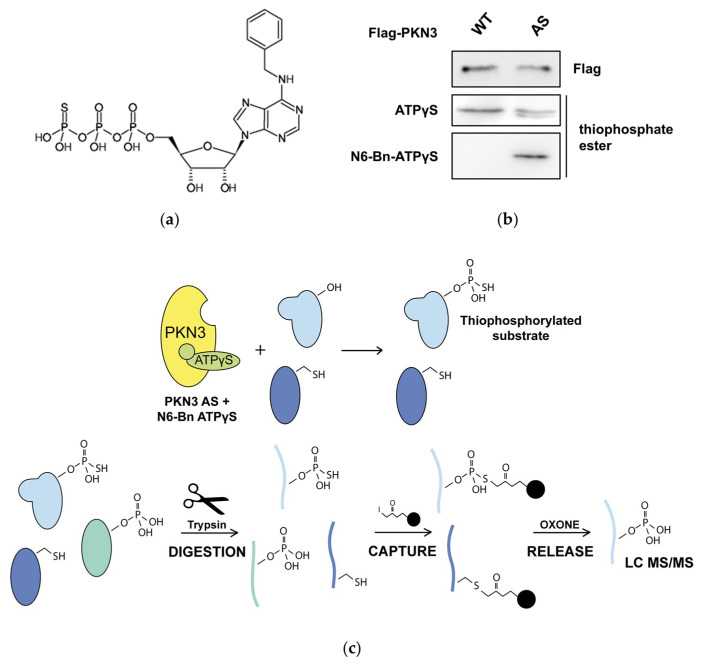
(**a**) Structural formula of N6-Benzyl ATPγS (N6-Bn ATPγS). (**b**) GST-fused peptide derived from GSK3 was used as a substrate in a kinase reaction with either Flag-PKN3 (Protein Kinase N3) WT or AS (analog sensitive). ATPγS or N6-Bn ATPγS were used as cofactors for phosphorylation. Thiophosphorylated substrates were alkylated with PNBM (*p*-nitrobenzyl mesylate) and immunoblotted with anti-thiophosphate ester antibody. (**c**) An outline of the phosphoproteomic screen: PKN3 AS thiophosphorylates its substrates using N6-Bn ATPγS in a lysate of MDA-MB-231 cells. Proteins are denatured and digested by trypsin. Thiol-containing peptides are captured by iodoacetyl beads and after oxidation only phosphopeptides are released and analyzed by LC MS/MS (liquid chromatography with tandem mass spectrometry).

In order to identify new PKN3 substrates we decided to use an analog-sensitive mutant of PKN3 (PKN3 AS). This approach is based on mutation of the gatekeeper residue allowing the kinase to use synthetic ATP analogs with a bulky group in the N6 position, thus providing specificity to the screen [16]. Therefore, we designed and created PKN3 AS by substitution of Thr639 for glycine. Given the high structural similarity of PKN3 with PKC kinases we chose N6-Benzyl ATPγS (N6-Bn ATPγS) (Figure 1a) for our studies since two of the PKC kinases with a mutation in gatekeeper residue have already been shown to effectively use it [16,31]. To test whether PKN3 AS is able to use N6-Bn ATPγS, we performed a kinase reaction with GST-fused peptide derived from GSK3 in the presence of either ATPγS or N6-Bn ATPγS. Thiophosphorylated substrates were then treated with alkylation agent PNBM (*p*-nitrobenzyl mesylate) and immunoblotted with an antibody recognizing thiophosphate-ester (clone 51-8). As expected, both PKN3 WT and PKN3 AS were able to use ATPγS but N6-Bn ATPγS could only be used by PKN3 AS (Figure 1b). The phosphoproteomic screen was performed in the lysate of MDA-MB-231 breast cancer cells expressing either PKN3 AS or PKN3 KD (kinase dead) as a control. After the reaction, the samples were denatured, digested and the thiol containing peptides were captured using iodoacetyl beads. The phosphopeptides released after the oxidation with Oxone were analyzed by LC MS/MS (liquid chromatography with tandem mass spectrometry) (Figure 1c). The list of the 20 highest-scoring targets with putative PKN3 phosphorylation site is shown in Table 1 (complete list is presented in Supplementary Table S1). We subjected the list of identified putative substrate proteins to GO (gene ontology) enrichment analysis using ShinyGO [32], however, no significant enrichment was found on the FDR (false discovery rate) level of 0.05. 

Since PKN3 is a kinase acting downstream of active Rho GTPases and was shown previously to interact with two RhoGAP proteins and phosphorylate them (GRAF1, GRAF2) [33], we decided to choose ARHGAP18 for validation of the screen and further study.

### 2.2. ARHGAP18 is Phosphorylated by PKN3

**Figure 2:**
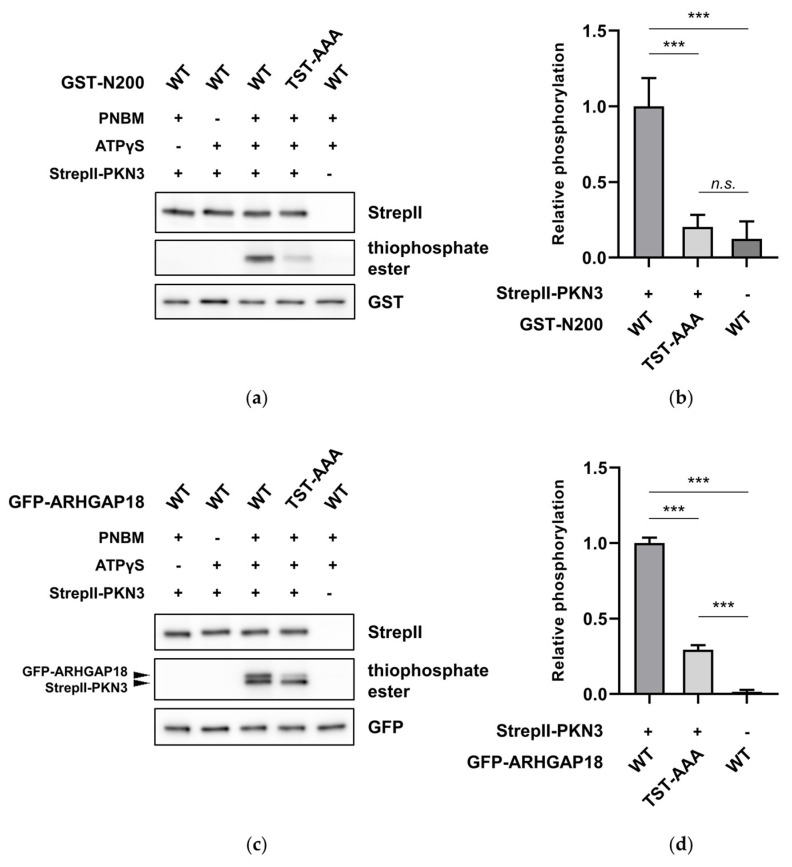
PKN3 phosphorylates ARHGAP18 (Rho GTPase Activating Protein 18) on Thr154, Ser156 and Thr158. An in vitro kinase reaction of WT and unphosphorylatable TST-AAA (Thr154, Ser156 and Thr158 to alanine) variants of (**a**) GST-fused fragment of ARHGAP18 comprising the first 200 amino acids or (**c**) the full-length GFP-ARHGAP18 (upper band; lower band corresponds to StrepII-PKN3 autophosphorylation) in the presence of StrepII-PKN3 and ATPγS. Thiophosphorylated substrates were treated with PNBM and immunoblotted using anti-thiophosphate ester antibody. Reactions with ATPγS or PNBM only were used as specificity controls. Quantification of relative phosphorylation from three independent experiments is shown for both (**b**) GST-N200 and (**d**) full-length GFP-ARHGAP18 variants. Statistical analysis was performed using ANOVA with Tukey′s multiple comparison test: *** *p* ≤ 0.001, n.s.—not significant.

In the phosphoproteomic screen we identified two phosphorylated residues in the sequence of ARHGAP18 – Ser156 and Thr158. Interestingly, we noticed that although phosphorylation of Thr154 did not appear in our phosphoproteomic results, the region surrounding this residue strongly resembles the PKN3 phosphorylation consensus motif [34], mainly due to the presence of a preferred arginine residue in the position ₋3, suggesting it could be also phosphorylated by PKN3. To validate our results and hypothesis, we fused the first 200 amino acids of ARHGAP18 to GST (GST-N200), substituted Thr154, Ser156 and Thr158 for unphosphorylatable alanine (GST-N200 TST-AAA) and subjected to in vitro kinase reaction. Since there are no phospho-specific antibodies available for ARHGAP18 we used ATPγS as a cofactor for phosphorylation with subsequent alkylation to detect PKN3-mediated phosphorylation as described above. As shown in Figure 2a, GST-N200 was readily phosphorylated by PKN3, however, the substitution of the candidate sites for alanine led to a significant reduction of phosphorylation (Figure 2a,b). In order to support our results, we further performed an in vitro kinase reaction also with the full-length GFP-fused variants of ARHGAP18 (WT and TST-AAA). As expected, PKN3 was able to phosphorylate also the full-length ARHGAP18 (upper band) and, importantly, phosphorylation of GFP-ARHGAP18 TST-AAA was reduced to a comparable extent as in GST-N200 (Figure 2c,d). These results support our data from phosphoproteomic screen and suggest that ARHGAP18 can be phosphorylated by PKN3 on Thr154, Ser156 and Thr158.

### 2.3. PKN3 Interacts with ARHGAP18 via its N-Terminal Region

**Figure 3:**
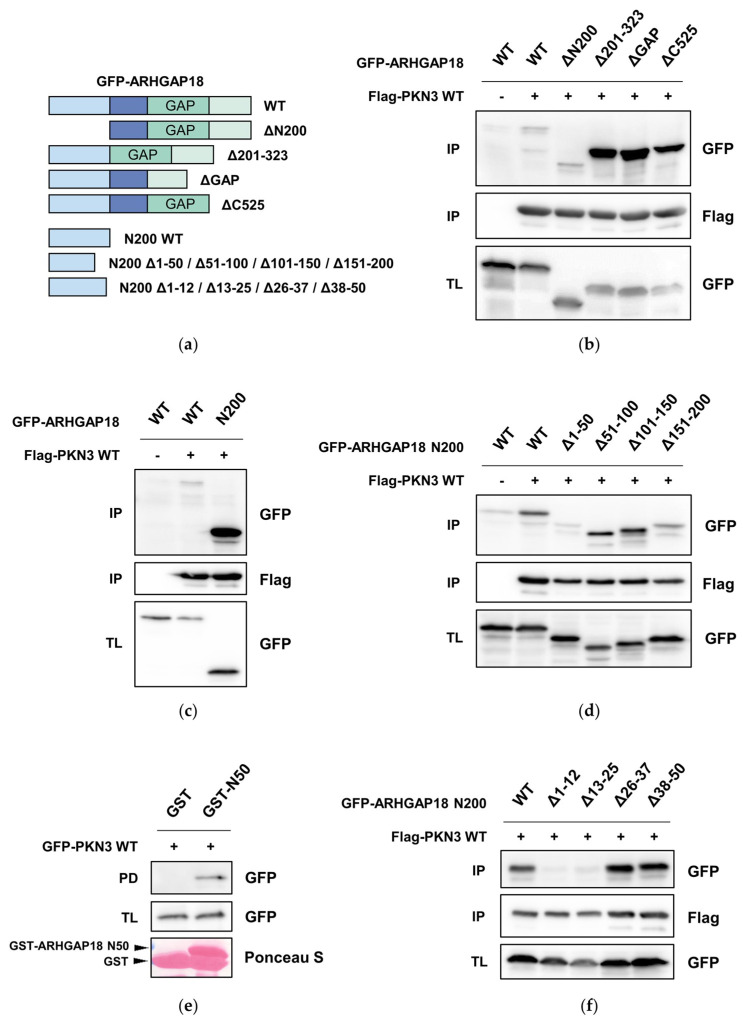
ARHGAP18 interacts with PKN3 (**a**) Schematic representation of ARHGAP18 mutants used throughout the study. (**b**–**d**,**f**) Co-immunoprecipitation experiments of indicated GFP-ARHGAP18 variants with Flag-PKN3. (**e**) Pull-down of GFP-PKN3 using either GST only or GST-fused fragment encompassing the first 50 amino acids of ARHGAP18 (GST-ARHGAP18 N50). All the samples were subjected to SDS-PAGE and immunoblotted with respective antibodies. GST-fused proteins were stained using Ponceau S staining. IP—immunoprecipitation; PD—pull-down; TL—total lysate.

PKN3 was shown to directly interact with two other Rho-GAP proteins from the Graf family via their SH3 domain [33]. Therefore, we next analyzed whether PKN3 and ARHGAP18 can also interact with each other. Since there is no SH3 domain in ARHGAP18 we decided to narrow down the potential interaction interface producing mutants of GFP-ARHGAP18 deleting either the first 200 amino acids (ΔN200), the region between amino acids 201 and GAP domain (Δ201-323), GAP domain (ΔGAP) or the *C*-terminal region following the GAP domain (ΔC525) (Figure 3a). Co-immunoprecipitation of individual deletion mutants with Flag-PKN3 indicated there is indeed an interaction between ARHGAP18 and PKN3. Surprisingly, GFP-ARHGAP18 Δ201-323, ΔGAP and ΔC525 exhibited very strong interaction with PKN3 when compared to full-length GFP-ARHGAP18 and ΔN200 (Figure 3b). This suggested that the interaction is mediated via the *N*-terminal part of ARHGAP18 and is inhibited by the following regions. To test this, we created a construct comprising only the first 200 amino acids fused to GFP (N200) and subjected to co-immunoprecipitation with Flag-PKN3. We observed a significant increase in binding of the GFP-ARHGAP18 N200 with PKN3 when compared to full-length GFP-ARHGAP18 (Figure 3c). To further specify the region of interaction we created deletion mutants of GFP-ARHGAP18 N200 sequentially lacking 50 amino acids: 1–50 (N200 Δ1–50), 51–100 (N200 Δ51–100), 101–150 (N200 Δ101–150) or 151–200 (N200 Δ151–200). Deletion of the first 50 amino acids (GFP-ARHGAP18 N200 Δ50) led to strong decrease of the binding to PKN3 suggesting that the binding sequence resides within this region (Figure 3d). To further confirm our results, we performed a pull-down of GFP-PKN3 with GST-bound fragment of ARHGAP18 containing only the first 50 amino acids (N50). As anticipated, we were able to pull-down GFP-PKN3 with GST-ARHGAP18 N50 but not with GST alone (Figure 3e). To finally pinpoint the binding sequence, we created deletion mutants of GFP-ARHGAP18 N200 lacking the amino acids 1–12, 13–25, 26–37 or 38–50 and subjected to co-immunoprecipitation. We found that both GFP-ARHGAP18 N200 Δ1–12 and Δ13–25 almost completely lost the ability to interact with PKN3 (Figure 3f). Taken together, these results suggest PKN3 is able to interact with ARHGAP18 and the first 25 amino acids of ARHGAP18 are necessary for the interaction.

### 2.4. Phosphorylation of ARHGAP18 Isoform1 but not Isoform2, Promotes Interaction with PKN3

**Figure 4:**
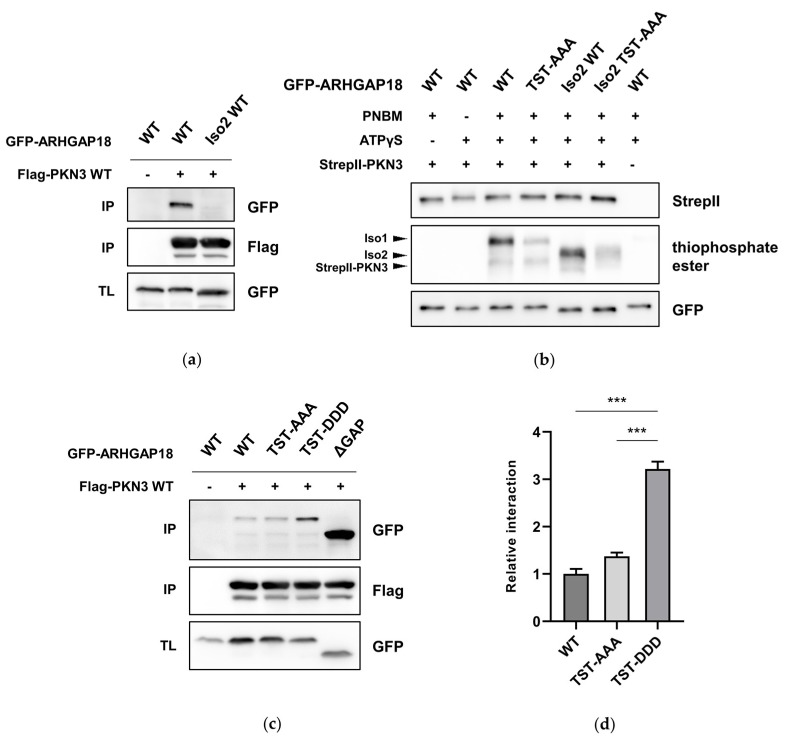

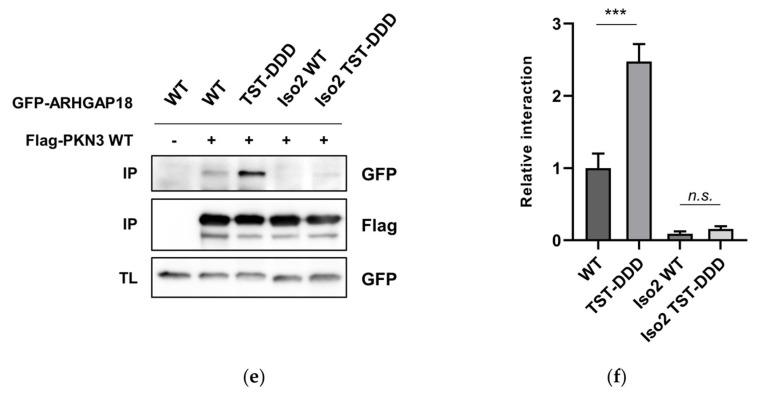
Differential effects of ARHGAP18 isoforms. (**a**) GFP-ARHGAP18 WT and Iso2 (isoform 2) WT were immunoprecipitated with Flag-PKN3 using Anti-Flag Affinity Gel. Samples were subjected to SDS-PAGE and immunoblotted with the respective antibodies. (**b**) An in vitro kinase reaction of WT and unphosphorylatable (TST-AAA) variants of both GFP-ARHGAP18 isoforms in the presence of StrepII-PKN3 and ATPγS. Thiophosphorylated substrates were treated with PNBM and immunoblotted using anti-thiophosphate ester antibody. Reactions with ATPγS or PNBM only were used as specificity controls. (**c**,**e**) Individual variants of GFP-ARHGAP18 were immunoprecipitated with Flag-PKN3. Samples were subjected to SDS-PAGE and immunoblotted using respective antibodies. (**d**,**f**) Quantification of three independent immunoprecipitation experiments is shown. Statistical analysis was performed using ANOVA with (**d**) Dunnett′s or (**f**) Tukey′s multiple comparison test: *** *p* ≤ 0.001; n.s.—not significant. IP—immunoprecipitation; TL—total lysate.

Two isoforms of ARHGAP18 have been described with the only difference between the two being, that isoform 2 (Iso2) is missing the first 45 amino acids [18,19]. Since we showed that the interaction of ARHGAP18 with PKN3 is predominantly mediated via the first 25 amino acids of ARHGAP18, we hypothesized there could be a difference in interaction of individual ARHGAP18 isoforms with PKN3. Therefore, we created GFP-ARHGAP18 Iso2 WT by deleting the first 45 amino acids and showed that, indeed, ARHGAP18 Iso2 almost completely lost the ability to interact with PKN3 (Figure 4a). Interestingly, despite the differences in the ability of individual ARHGAP18 isoforms to interact with PKN3, both isoforms can be phosphorylated by PKN3 to a similar extent (Figure 4b). To test whether ARHGAP18 phosphorylation could affect its interaction with PKN3, we created a phosphomimicking mutant of ARHGAP18 by substitution of Thr154, Ser156 and Thr158 for aspartate (TST-DDD). In a co-immunoprecipitation of either phosphomimicking (TST-DDD) or unphosphorylatable (TST-AAA) variant of ARHGAP18 with Flag-PKN3 we observed more than three-fold increase in the interaction of ARHGAP18 TST-DDD with PKN3 when compared to WT (Figure 4c,d). Finally, we showed that interaction with PKN3 was promoted only in case of TST-DDD but not Iso2 TST-DDD ARHGAP18 (Figure 4e,f). These results suggest PKN3 is able to phosphorylate both ARHGAP18 isoforms but only Iso1 exhibits increased interaction with PKN3 after phosphorylation.

### 2.5. Phosphorylation of ARHGAP18 Leads to Activation of Its GAP Domain

**Figure 5:**
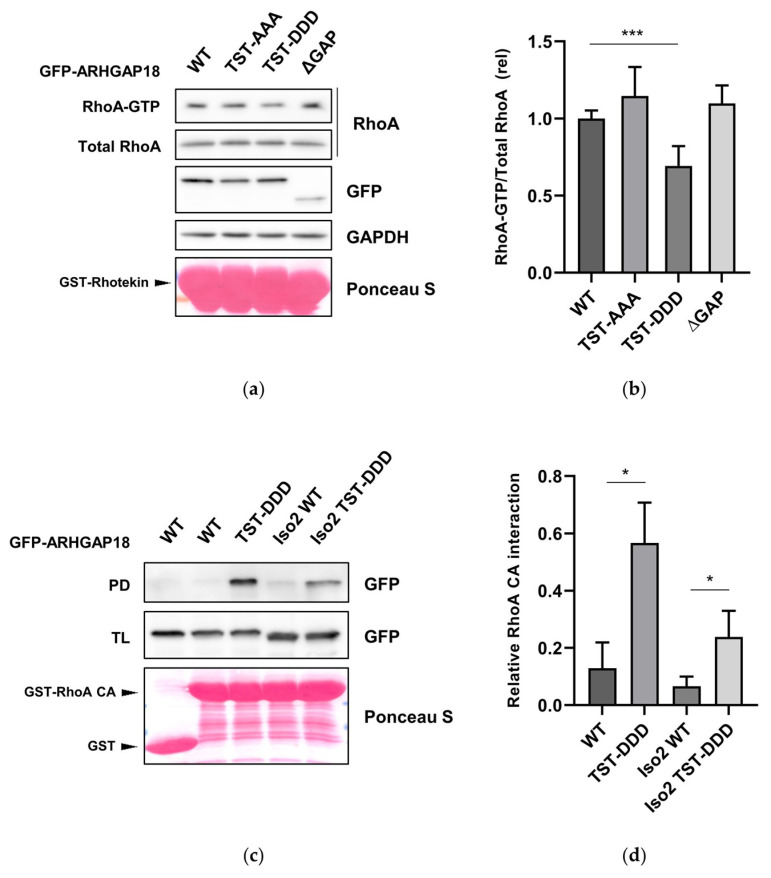
Phosphorylation of ARHGAP18 by PKN3 leads to activation of its GAP domain (**a**) U2OS cells expressing indicated variants of GFP-ARHGAP18 were subjected to pull-down of active RhoA using GST-fused Rhotekin. (**b**) Quantification of three independent RhoA-GTP pull-down experiments was performed. (**c**) Pull-down of individual GFP-ARHGAP18 variants using GSH-agarose-bound constitutively active RhoA (RhoA CA). (**d**) Quantification of three independent pull-down experiments using GST-RhoA CA is shown. All the samples were subjected to SDS-PAGE and immunoblotted with respective antibodies. GST-Rhotekin and GST-RhoA CA were stained by Ponceau S. Statistical analysis was performed using ANOVA with (**b**) Dunnett’s multiple comparison test: * *p* ≤ 0.05 and *** *p* ≤ 0.001. PD—pull-down; TL—total lysate.

To assess whether the phosphorylation of ARHGAP18 could have some effect on its function we first focused on the levels of active RhoA. U2OS cells expressing individual variants of GFP-ARHGAP18 were subjected to RhoA-GTP pull-down assay using immobilized GST-Rhotekin. Interestingly, a significant decrease in the levels of active RhoA was observed in cells expressing GFP-ARHGAP18 TST-DDD when compared to WT suggesting that phosphorylation of ARHGAP18 leads to activation of its GAP domain (Figure 5a,b). To support our results, we analyzed the effect of ARHGAP18 phosphorylation on the activation of its GAP domain by a pull-down assay using constitutively active RhoA (RhoA CA). We observed that the phosphomimicking variant of both ARHGAP18 isoforms displayed a substantial increase in interaction with RhoA CA when compaired to the corresponding WT (Figure 5c,d). Taken together, these data suggest phosphorylation of ARHGAP18 by PKN3 leads to activation of ARHGAP18 GAP domain resulting in decrease of the levels of active RhoA. 

## 3. Discussion

**Figure 6:**
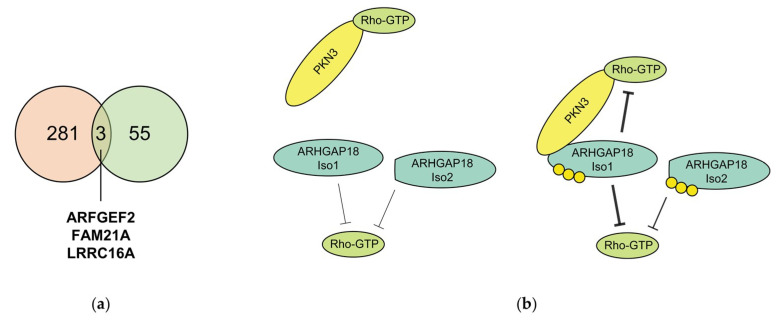
(**a**) Venn diagram representing the overlap between the proteins identified as putative PKN3 substrates in this study (orange) and the phosphoproteomic data presented in Browne et al., 2019 (green) [35]. Three genes were identified in both datasets: ARFGEF2, FAM21A and LRRC16A. (**b**) A model proposing PKN3-mediated regulation of ARHGAP18 activity. ARHGAP18 in its unphosphorylated form exhibits low GAP activity and its *N*-terminus is potentially sterically blocked and therefore inaccessible for interactions. ARHGAP18 phosphorylation (yellow circles) by PKN3 induces structural change leading to activation of GAP domain and in case of ARHGAP18 Iso1 also to great strengthening of ARGAP18 and PKN3 interaction mediated by the release of its *N*-terminus which becomes accessible for interaction with PKN3.

Although PKN3 is an important effector kinase of small Rho GTPases and a key player in regulation of processes such as cytoskeleton organization [4], proliferation and promotion of malignant growth of various cancer types [8,9,13], its downstream signaling remains largely understudied. In order to identify novel PKN3 substrates we performed a phosphoproteomic screen using analog sensitive PKN3. We identified 418 putative PKN3 phosphorylation sites in 281 proteins. Surprisingly, however, we found no significant functional enrichment in GO terms among the newly identified substrates. Recently, another screen for PKN3 substrates was performed using JZ128, a new selective covalent inhibitor of PKN3 in a novel chemoproteomic approach—CITe-Id (Covalent Inhibitor Target-site Identification) [35]. When we compared the two datasets, three proteins were identified in both of the screens – ARFGEF2, FAM21A and LRRC16A (Figure 6a). Both FAM21A, a component of the WASH complex and LRRC16A, also known as CARMIL, were shown to play a role in regulation of actin remodeling [36,37,38], highlighting the possible signaling crosstalk with PKN3. It is also surprising that proteins previously reported to be phosphorylated by PKN3, such as GRAF1, GRAF2 or BCAR1 [13,33] were not identified as PKN3 substrates in any of the screens. 

Among the identified putative substrates of PKN3 we selected ARHGAP18 for further study and validation. In an in vitro kinase reaction using either truncated or full-length protein we confirmed that PKN3 is able to phosphorylate ARHGAP18 on Thr154, Ser156 and Thr158. Although there are 80 serine and threonine residues present in the 663 amino acids-long sequence of ARHGAP18, mutation in these three candidate sites alone was sufficient to substantially impair the phosphorylation of ARHGAP18 by PKN3, suggesting the candidate sites are phosphorylated in a highly specific-manner. However, since the decrease in phosphorylation was not complete, we expect there are another residues in the sequence of ARHGAP18 phosphorylated by PKN3. We next found that PKN3 is able to interact with ARHGAP18 and we mapped the interaction interface to the first 25 amino acids in the sequence of ARHGAP18. Importantly, this region is missing in ARHGAP18 Iso2 which is translated from an alternative downstream start codon [18,19]. Interestingly, although we observed differences in interaction of PKN3 with individual isoforms of ARHGAP18, both isoforms were phosphorylated to a similar extent. It is notable, that phosphorylation of several RhoGAP proteins was reported to have different effects on their function [39,40,41]. Recently, we have described an activating phosphorylation of ARHGAP42 Tyr376 by Src kinase [42]. Similarly, we found that phosphorylation of ARHGAP18 by PKN3 on Thr154, Ser156 and Thr158 leads to activation of its GAP domain, thus decreasing the levels of active RhoA.

Notably, PKN3 and ARHGAP18 share a lot of similar traits in the signaling of endothelial cells. After downregulation of either ARHGAP18 or PKN3 in endothelial cells, the cells lose the capacity of characteristic tube formation in both 2D and 3D environment [4,10,28] and exhibit disrupted cell junctions [10,18]. Recently, the role of ARHGAP18 has been extensively studied in the context of atherosclerosis, a chronic inflammatory disease of the arteries [43]. ARHGAP18 was shown to act as an anti-inflammatory and athero-protective gene that facilitates flow-responsive endothelial cell alignment via ARHGAP18/YAP axis [29,30]. Interestingly, PKN3 was identified in a module of genes associated to transendothelial migration of leukocytes leading to coronary artery disease [44]. Moreover, depletion of PKN3 was shown to attenuate pro-inflammatory activation of endothelial cells caused by defects in glycosylation of ICAM-1 adhesion molecules, suggesting its potential involvement in the promotion of atherosclerosis [10,15]. Finally, PKN3 has been recently demonstrated to play a role in bone resorption downstream of non-canonical Wnt5a/ Ror2 signaling cascade [5,6] that regulates the secretion of pro-inflammatory cytokines necessary for atherosclerosis development [45]. All these findings highlight potential crosstalk of ARHGAP18 and PKN3 and should be considered in the future research.

Based on our results we propose a model, where ARHGAP18 in its unphosphorylated form exhibits low GAP activity and its *N*-terminus is potentially sterically blocked and, therefore, inaccessible for interactions. ARHGAP18 phosphorylation induces structural change leading to activation of GAP domain and in case of ARHGAP18 Iso1 also to a great strengthening of ARHGAP18 and PKN3 interaction mediated by the release of its *N*-terminus, which becomes accessible for interaction with PKN3. Our data also suggest that phosphorylation of both ARHGAP18 isoforms could facilitate a negative feedback loop in regulation of signaling mediated by Rho GTPases. In case of ARHGAP18 Iso1 this feedback mechanism is further strengthened by formation of a ternary complex between ARHGAP18, PKN3 and Rho GTPases (Figure 6b).

Taken together, based on the results of our phosphoproteomic screen we identified ARHGAP18 as a new PKN3 substrate and interaction partner. We showed that phosphorylation of ARHGAP18 by PKN3 leads to activation of its GAP domain and contributes to regulation of active RhoA levels, implying the possible crosstalk of PKN3 and ARHGAP18 signaling in cancer and other diseases. We also believe the results of our screen will serve as a basis for better understanding of PKN3 signaling and its future study. 

## 4. Materials and Methods 

### 4.1. Cell Lines and Cell Cultivation

All the cell lines were cultured in DMEM (Sigma, Piscataway, NJ, USA) supplemented with 10% FBS (Sigma, Piscataway, NJ, USA) and 10 µg/mL ciprofloxacin (Sigma, Piscataway, NJ, USA) in humidified incubator with 5% CO_2_. U2OS cells were purchased from ATCC (#HTB-96), MDA-MB-231 cells were obtained from Dr. Zadinová as described previously [13]. Unless otherwise stated, all the experiments were performed using MDA-MB-231 cells. 

### 4.2. Plasmid Construction

Human Flag-PKN3 WT and KD (kinase dead) in pcDNA3, as well as StrepII-PKN3 in pcDNA3 were used previously [13]. Analog-sensitive (AS, T639G) PKN3 was designed based on a prediction of gatekeeper residue as described in Hertz et al. [17] and created using Q5^®^ Site-Directed Mutagenesis Kit (New England Biolabs, Ipswich, MA, USA) following the manufacturer’s instructions with the corresponding primers (T639G F/ R) listed in Supplementary Table S2.

cDNA encoding human ARHGAP18 isoform 1 was newly synthesized using GeneArt Gene Synthesis (Thermo Scientific, Waltham, MA, USA) (sequence shown in Supplementary Table S3). Silent mutations were introduced in the design of ARHGAP18 cDNA in order to disrupt *XhoI*, *SacI*, *BamHI* and *NcoI* restriction sites. Synthesized sequence was cloned into pEGFP c1 vector using *BglII* and *EcoRI* sites. ARHGAP18 N200 constructs were created by PCR amplification with respective primers (ARHGAP18 N200 F/R), digested with *EcoRI/BamHI* and cloned into pEGFP c1 via *EcoRI/BglII* sites and into pGEX 2T bacterial expression vector via *EcoRI*/*BamHI* sites. All the mutants of ARHGAP18 WT or N200 (Iso2 WT, ΔN200, Δ201-323, ΔGAP, ΔC525, TST-AAA, TST-DDD, Iso2 TST-DDD and Iso2 TST-AAA) in either pEGFP c1 or pGEX 2T were created by whole plasmid synthesis approach (WHOPS) with Pfu-X7 polymerase and subsequent *DpnI* treatment with the respective primers listed in the Supplementary Table S2. The deletion variants of ARHGAP18 N200 (Δ1–50, Δ51–100, Δ101–150, Δ151–200, Δ1–12, Δ13–25, Δ26–37, Δ38–50) were created using WHOPS using the primers listed in the Supplementary Table S2 and ARHGAP18 N200 WT pEGFP c1 as a template. GST-RHG18 N50 construct was created by PCR amplification with ARHGAP18 N200 F and ARHGAP18 N50 R primers and cloned into pGEX 2T bacterial expression vector via *EcoRI*/*BamHI* sites. All the created constructs were verified by sequencing. 

### 4.3. Protein Expression and Purification

GST alone and GST-fused proteins (GST-GSK3 peptide, GST-ARHGAP18 N200 WT/TST-AAA, GST-Rhotekin, GST-RhoA CA (G14V)), were purified using BL21 (DE3) *E. coli* strain. Briefly, cells were grown in 1.5 × LB medium (Duchefa Biochemie, Haarlem, The Netherlands) and cultured to 0.8 OD_595_. IPTG was added to the final concentration of 0.4 mM and incubated overnight at room temperature. Proteins were purified from cleared lysates using Pierce^®^ Glutathione Agarose (Thermo Scientific, Waltham, MA, USA).

### 4.4. Screen for PKN3 Substrates, Sample Preparation and Data Analysis

The screen was performed following the protocol published by Hertz and colleagues [17]. Briefly, lysates of MDA-MB-231 cells transfected with either PKN3 AS or PKN3 KD were prepared using 1% Triton X-100 (Sigma, Piscataway, NJ, USA) in TBS (50 mM Tris-HCl, pH 7.1 (20 °C), 150 mM NaCl), sonicated and cleared by centrifugation. For each sample, 3 mg of total protein was used and kinase reactions were incubated for 40 min at room temperature in the presence of 200 µM ATP (Sigma, Piscataway, NJ, USA), 3 mM GTP (Sigma, Piscataway, NJ, USA) and 200 µM N^6^-benzyl-ATPγS (Jena Bioscience, Jena, Germany). Afterwards, denaturation buffer (8 M Urea (Sigma, Piscataway, NJ, USA), 10 mM TCEP (Sigma, Piscataway, NJ, USA), 100 mM NH_4_HCO_3_ (Sigma, Piscataway, NJ, USA), 2 mM EDTA (Sigma, Piscataway, NJ, USA)) was added to the samples to 6 M final concentration of Urea, incubated for 1 h at 55 °C and cooled to RT for 10 min. Samples were diluted using 50 mM NH_4_HCO_3_ to a 2 M final concentration of Urea and 1 M TCEP was added to final concentration of 10 mM. 50 µg of Trypsin (Thermo Scientific, Waltham, MA, USA) was added to each sample and incubated overnight in 37 °C with gentle agitation. Samples were then acidified to the final concentration of 0.1% TFA (Sigma, Piscataway, NJ, USA) and peptides were extracted using Oasis^®^ PRiME HLB columns (Waters, Milford, MA, USA). Peptides washed with 0.1% TFA in water were eluted with 1 mL of 0.1% TFA in 50% Acetonitrile in water and concentrated to 50 µL using speed vacuum. For capture of thiophosphorylated peptides, 100 µL of UltraLink^®^ Iodoacetyl beads (Thermo Scientific, Waltham, MA, USA) per sample was washed with 200 mM HEPES pH 7.0 and blocked for 10 min in dark with 5 µL of 5 mg/mL BSA (Sigma, Piscataway, NJ, USA) in 50% Acetonitrile 50% 20 mM Hepes pH 7.0. Samples adjusted to a final concentration of 20 mM HEPES pH 7.0 and 50% Acetonitrile were added to the beads and incubated in dark place overnight at RT. After incubation, the beads were washed in the following order with 1 mL of water, 5 M NaCl, 50% Acetonitrile, 5% Formic Acid and incubated in 10 mM DTT for 10 min. Finally, samples were eluted for 10 min in 1 mg/mL OXONE (Sigma, Piscataway, NJ, USA) pH 3.5, desalted with ZipTip and analyzed by Thermo Orbitrap Fusion coupled with Thermo Ultimate 3000 HPLC. Raw data were analyzed using MaxQuant software with MaxLFQ algorithm and the MS/MS spectra were searched against Uniprot-SwissProt human database both in forward and reverse using Andromeda search engine. Search parameters were set to standard trypsin digestion with two missed cleavages, variable *N*-terminal carbamylation, variable methionine oxidation and variable serine/threonine phosphorylation with the maximum number of modifications per peptide set to 5. The identified peptides were filtered based on 1% FDR. For selection of putative PKN3 phosphorylation sites, all the phosphosites identified in the control samples were eliminated from the analysis, together with phosphopeptides with localization probability below the cutoff value 0.75.

### 4.5. Immunoprecipitation and Immunoblotting

MDA-MB-231 cells were transfected using PEI (Polysciences, Inc.,Warrington, PA, USA). After 48 h, the cells were washed with ice-cold PBS, lysed using 1% Triton X-100 in TBS (50 mM Tris-HCl, pH 7.1 (20 °C), 150 mM NaCl) and the lysates were cleared by centrifugation. Subsequently, 20 µL of Anti-Flag Affinity Gel (Bimake, Houston, TX, USA) was added to each lysate and rotated for 3 h in 4 °C. After the incubation, the beads were washed twice with ice-cold lysis buffer, once with TBS and resuspended in SDS-PAGE sample buffer. After the separation of the samples using gradient SDS polyacrylamide gels (6–15%), proteins were transferred to Amersham Protran 0.45 µm nitrocellulose membrane (GE Healthcare, Chicago, IL, USA) using Transblot Turbo (Bio-Rad Laboratories, Hercules, California, CA, USA) in a buffer containing 300 mM Tris, 300 mM Glycine, 0.025% SDS and 20% EtOH. After the transfer, the membranes were stained in Ponceau S (Sigma, Piscataway, NJ, USA) for total protein, washed in TBS and blocked with 4% BSA (Sigma, Piscataway, NJ, USA) in TBS for 30 min in RT. The membranes were incubated with respective antibodies diluted in 1% BSA (Sigma, Piscataway, NJ, USA) in TBST overnight in 4 °C. The antibody against thiophosphate ester (clone 51-8, Abcam, Cambridge, UK) was diluted 1:5000 in 5% milk in TBST. Secondary antibodies were diluted in 2% milk in TBST. The membranes were developed with either WesternBright ECL (Advansta, San Jose, CA, USA) or SuperSignal™ West Femto Maximum Sensitivity Substrate (Thermo Scientific, Waltham, MA, USA) using Amersham Imager 600 (GE Healthcare, Chicago, IL, USA).

### 4.6. Antibodies

StrepII Tag antibody (clone 517, #NBP2-43735) was purchased from Novus Biologicals. Anti-thiophosphate ester antibody (clone 51-8, #ab133473) and GFP antibody used for immunoblotting (#ab290) were purchased from Abcam (Cambridge, UK). RhoA antibody (clone 67B9, #2117) was purchased from Cell Signaling Technology. GFP antibody used for immunoprecipitation (clone 3E6, #A-11120) was purchased from Invitrogen. Flag tag monoclonal antibody (clone M2, #F1804) and GST antibody (#G7781) were purchased from Sigma.

### 4.7. Kinase Assays

Kinase assays were performed as described previously [13]. Briefly, StrepII-PKN3-transfected MDA-MB-231 cells were lysed using 1% Triton X-100 in TBS (50 mM Tris-HCl, pH 7.1 (20 °C), 150 mM NaCl) supplemented with 10 mM Glycerol-2-Phosphate (Sigma, Piscataway, NJ, USA) and inhibitors of proteases and phosphatases (1:100 each, Bimake, Houston, TX, USA). The kinase was precipitated from the cleared lysate for 3 h in 4 °C using Strep-Tactin^®^ Superflow^®^ resin (IBA Lifesciences, Göttingen, Germany) and eluted with 1.25× Buffer E (Strep-Tactin Elution Buffer, IBA Lifesciences, Göttingen, Germany).

As a positive control for testing of PKN3 AS, a peptide derived from GSK3 was used [9]. GST-fused *N*-terminal fragments of ARHGAP18 (N200 WT, N200 TST-AAA) were produced and purified as described above (see Protein expression and purification). For the kinase assays of full-length ARHGAP18 proteins, individual GFP-ARHGAP18 variants (WT, TST-AAA, Iso2 WT, Iso2 TST-AAA) were immunoprecipitated from transiently transfected U2OS cells. Lysates were prepared with the lysis buffer containing 1% Triton X-100 in TBS as described above and proteins were precipitated using anti-GFP 3E6 antibody (Invitrogen) and Protein A Sepharose 4 Fast Flow (GE Healthcare, Chicago, IL, USA). Immobilized proteins were eluted using 0.1 M Glycine pH 3.5 for 10 min in RT, corresponding volume of 1 M Tris pH 9.2 was added to adjust the pH to 7.5 and eluted proteins were used as a substrate in kinase reaction. 

All the kinase reactions were carried out in the kinase buffer containing 30 mM Tris pH 7.5, 4 mM MgCl_2_ (Sigma, Piscataway, NJ, USA), 10 mM Glycerol-2-Phosphate (Sigma, Piscataway, NJ, USA) and 5 mM DTT (Sigma, Piscataway, NJ, USA). N^6^-benzyl-ATPγS or ATPγS were used as indicated in the final concentration of 1 mM. Reactions were incubated for 45 min in 35 °C and stopped by adding 0.5 mM EDTA pH 8.0 to the final concentration of 20 mM. Thiophosphorylated proteins were then alkylated with 50 mM PNBM (*p*-Nitrobensyl mesylate) (Abcam, Cambridge, UK) at RT for 2 h, subjected to SDS-PAGE and immunoblotted using anti-thiophosphate ester antibody (clone 51-8, Abcam, Cambridge, UK). 

### 4.8. RhoA-GTP Pull-Down Assay

For the analysis of active RhoA, U2OS cells were transfected with individual variants of GFP-ARHGAP18 (WT, TST-AAA, TST-DDD, ΔGAP) or GFP alone, starved for 3 h and then lysed in 1% Triton X-100 in TBS (50 mM Tris-HCl, pH 7.1 (20 °C), 150 mM NaCl) with inhibitors of proteases and phosphatases (1:100, Bimake, Houston, TX, USA) after 5 min stimulation by DMEM with 10% FBS. The lysates were centrifuged (13,000× *g*, 13 min) and the supernatants were equalized for total protein (DC™ Protein Assay, Bio-Rad, Hercules, California, CA, USA). Agarose-bound GST-Rhotekin (20 µg) was added to each lysate and rotated for 45 min in 4 °C. Afterwards, the beads were washed twice with ice-cold lysis buffer, once with TBS and resuspended in SDS-PAGE sample buffer. The samples were subjected to SDS-PAGE and immunoblotted with respective antibodies as described above. 

### 4.9. RhoA Pull-Down Assay

The RhoA pull-down assay was performed using purified constitutively active form of RhoA (RhoA-CA, G14V) as described in García-Mata et al. [46]. Cells transfected with individual variants of GFP-ARHGAP18 were washed twice with ice-cold HBS (20 mM HEPES pH 7.5, 150 mM NaCl) and lysed in HBS containing 1% Triton X-100, 5 mM MgCl_2_ (Sigma, Piscataway, NJ, USA) and 1mM DTT supplied with inhibitors of proteases (1:100, Bimake, Houston, TX, USA) and phosphatases (1:100, Bimake, Houston, TX, USA). The lysates were equalized for the total amount of GFP signal. Agarose-bound RhoA-CA (15 μg) was added to each lysate and rotated for 1 h at 4 °C. The beads were washed three times with lysis buffer and resuspended in SDS-PAGE sample buffer. The samples were subjected to SDS-PAGE and analyzed by immunoblotting as described above.

## Figures and Tables

**Table 1:** The list of 20 putative PKN3 substrates with the highest identification score. Surrounding sequence of +/−8 amino acids around the identified phosphosite is shown. PLP—phosphosite localization probability.

Gene	Site	Surrounding Sequence	PLP	Score
ARHGAP18	T158	KRVETVSQTLRKKNKQY	1.00	111.46
	S156	VQKRVETVSQTLRKKNK	1.00	111.46
BRD4	S601	SKPPPTYESEEEDKCKP	1.00	101.9
CCDC144A	S805	EMARKKMNSEISHRHQK	0.99	82.029
CEACAM16	T402	NLTDTGRYTLKTVTVQG	1.00	100.25
	T398	VQKLNLTDTGRYTLKTV	1.00	100.25
DCAF12L1	S8	_MAQQQTGSRKRKAPAV	1.00	83.801
DSCAM	S1408	LPGDNGGSSIRGYILQY	0.98	87.498
IGSF22	S318	LSVGDKRMSAELTVLDE	0.99	84.817
KIF27	S754	TGNDAKSVSKQYSLKVT	1.00	120.77
	T746	DLIKELIKTGNDAKSVS	0.99	120.77
NFE2L2	T137	KLHHNYKITIYSM____	1.00	99.139
ODF1	S10	AALSCLLDSVRRDIKKV	1.00	103.7
OR4K15	T158	ICKPLHYMTVMSRRVCV	0.99	79.906
POLE1	S891	VKKPKVTISYPGAMLNI	1.00	82.483
	T889	TNVKKPKVTISYPGAML	1.00	82.483
PRDM16	S1218	DVLNSTLDSEALKHTLC	0.99	96.371
RETSAT	S322	VLTKATVQSVLLDSAGK	0.98	99.53
SACS	S1222	GIFTKPSLSAVLKHFKI	1.00	86.512
SPATA31D1	T1339	VLGSKSSPTLKTQPPPE	0.87	86.189
USP42	T786	PRDPGTPATKEGAWEAM	1.00	93.096
UTP20	S926	HLQVFSKFSNPRALYLE	1.00	101.65
VPS33B	S476	AGKITDAFSSLAKRSNF	0.78	83.206
	S477	GKITDAFSSLAKRSNFR	0.78	83.206
VWA3B	T99	EDGRVYNLTAKSELIYQ	1.00	84.244

## Supplementary Materials

Supplementary materials can be found at https://www.mdpi.com/1422-0067/21/20/7769/s1. Table S1: A complete list of putative PKN3 substrates identified in the phosphoproteomic screen; Table S2: The list of primers used in the study; Table S3: The sequence of synthesized ARHGAP18.

## Abbreviations

PKN3	Protein Kinase N3
ARHGAP18	Rho GTPase Activating Protein 18
N6-Bn ATPγS	N6-Benzyl ATPγS
PNBM	*p*-nitrobenzyl mesylate
