# Supplementary material for: A Screen for PKN3 Substrates Reveals an Activating Phosphorylation of ARHGAP18
Source: Int J Mol Sci. 2020 Oct 20;21(20):7769. doi: 10.3390/ijms21207769 (PMC7594087; doi:10.3390/ijms21207769)
Supplement: Supplementary file 1 [file ijms-21-07769-s001.zip › Supplementary material.pdf]

Supplementary table 2. The list of primers used in the study.

Primer name	Sequence
PKN3 T639G F	TTTGTGGGTGAGTTTGTGCC
PKN3 T639G R	GCAGGCATGGCTGGAGGT
ARHGAP18 N200 F	TAGGATCCATGAGCTGGCTCTCCAGT
ARHGAP18 N200 R	TAGAATTCTCATTTGTTTTCATTTGTTCT
ARHGAP18 N50 R	TAGAATTCTCAGCTTTCCTGGTTCATAGT
ARHGAP18 Iso2 F	ACAAGTCCGGACTCAGATCTATGAACCAGGAAAGCACCACCA
ARHGAP18 Iso2 R	TGGTGGTGCTTTCCTGGTTCATAGATCTGAGTCCGGACTTGT
ARHGAP18 ΔN200 F	ATCTATGAGCTGGTACCAAGGAAGAGA
ARHGAP18 ΔN200 R	TCTCTTCCTTGGTACCAGCTCATAGAT
ARHGAP18 Δ201-323 F	CAAATGAAAACAAAGTTCCATTGACAGC
ARHGAP18 Δ201-323 R	GCTGTCAATGGAACTTTGTTTTCATTTG
ARHGAP18 ΔGAP F	CTGGTCTTTTTTGCCCCAAGTTTATTGT
ARHGAP18 ΔGAP R	ACAATAAACTTGGGGCAAAAAAGACCAG
ARHGAP18 ΔC525 F	AACTTCTGTGGACATAGAACTCGAGAATTC
ARHGAP18 ΔC525 R	GAATTCTCGAGTTCTATGTCCACAGAAGTT
ARHGAP18 N200 Δ1-50 F	CGGACTCAGATCCATGACCACCATCAAAGTTA
ARHGAP18 N200 Δ1-50 R	TAACTTTGATGGTGGTCATGGATCTGAGTCCG
ARHGAP18 N200 Δ51-100 F	TATGAACCAGGAAAGCGTCAAAGAGCCTGATG
ARHGAP18 N200 Δ51-100 R	CATCAGGCTCTTTGACGCTTTCCTGGTTCATA
ARHGAP18 N200 Δ101-150 F	TCAAGAGGTGGTTGTTCGAGTAGAGACGGTCT
ARHGAP18 N200 Δ101-150 R	AGACCGTCTCTACTCGAACAACCACCTCTTGA
ARHGAP18 N200 Δ151-200 F	AGCAGCAGTTCAGAAGTGAGAATTCTGCAGTC
ARHGAP18 N200 Δ151-200 R	GACTGCAGAATTCTCACTTCTGAACTGCTGCT
ARHGAP18 N200 Δ1-12 F	CGGACTCAGATCCATGACAGCCTACCACCCCA
ARHGAP18 N200 Δ1-12 R	TGGGGTGGTAGGCTGTCATGGATCTGAGTCCG
ARHGAP18 N200 Δ13-25 F	CCAGGGAGTGGTACTAAACAGCCATGCAAAG
ARHGAP18 N200 Δ13-25 R	CTTTGCATGGCTGTTTAGTACCACTCCCTGG
ARHGAP18 N200 Δ26-37 F	GGACCAGACCGTCGGGAGTCGCAGATATGGCC
ARHGAP18 N200 Δ26-37 R	GGCCATATCTGCGACTCCCGACGGTCTGGTCC
ARHGAP18 N200 Δ38-50 F	GGAGGAAGCCACGTCGACCACCATCAAAGTTA
ARHGAP18 N200 Δ38-50 R	TAACTTTGATGGTGGTCGACGTGGCTTCCTCC

Supplementary table 3. The sequence of synthesized ARHGAP18. Silent mutations were introduced in the design of ARHGAP18 cDNA in order to disrupt *XhoI*, *SacI*, *BamHI* and *NcoI* restriction sites.

**ARHGAP18**
TACAGATCTATGAGCTGGCTCTCCAGTTCCCAGGGAGTGGTACTAACAGCCTACCACCCCAGCGGCAAGGACC<br>AGACCGTCGGGAACAGCCATGCAAAGGCAGGGGAGGAAGCCACGTCGAGTCGCAGATATGGCCAGTACACTAT<br>GAACCAGGAAAGCACCACCATCAAAGTTATGGAGAAGCCTCCATTTGATCGATCAATTTCCCAGGATTCTTTG<br>GATGAACTATCTATGGAAGACTATTGGATAGAACTAGAAAACATCAAGAAATCTAGTGAAAACAGCCAAGAAG<br>ATCAAGAGGTGGTTGTTGTCAAAGAGCCTGATGAGGGAGAATTGGAAGAAGAGTGGCTTAAAGAGGCCGGTTT<br>ATCCAATCTCTTCGGAGAGTCTGCTGGAGATCCACAGGAAAGCATTGTGTTTTTATCAACATTGACGCGGACC<br>CAGGCAGCAGCAGTTCAGAAGCGAGTAGAGACGGTCTCCCAGACCTTGAGGAAAAAAAACAAACAGTACCAGA<br>TTCCTGACGTCAGAGACATATTTGCTCAACAGAGAGAATCAAAAGAAACAGCTCCAGGTGGCACTGAATCGCA<br>GTCACTTAGAACAAATGAAAACAAATACCAAGGAAGAGATGACGAGGCATCTAACCTTGTTGGTGAAGAGAAG<br>CTGATCCCACCTGAGGAGACGCCTGCCCCTGAAACAGACATCAACCTGGAGGTATCATTTGCCGAGCAAGCAC<br>TCAATCAGAAAGAGAGTTCCAAGGAGAAAATCCAGAAGAGCAAAGGCGATGATGCCACATTACCTAGTTTCAG<br>ATTGCCAAAAGACAAAACGGGTACCACAAGGATTGGTGACCTCGCACCCCAGGACATGAAGAAAGTTTGCCAT<br>TTAGCCCTAATTGAGCTGACTGCCCTCTATGATGTATTGGGTATTGAGCTGAAACAACAAAAAGCTGTGAAAA<br>TCAAAACAAAAGATTCTGGTCTTTTTTGCGTTCCATTGACAGCGCTATTAGAACAAGATCAGAGGAAAGTACC<br>AGGAATGCGAATACCCTTGATCTTTCAAAAACTGATTTCTCGAATTGAAGAGAGAGGTTTGGAAACAGAAGGC<br>CTCTTACGAATCCCTGGAGCTGCCATTAGAATCAAGAATCTTTGCCAAGAACTAGAAGCAAAGTTTTATGAAG<br>GGACTTTTAATTGGGAAAGTGTCAAACAGCATGATGCCGCCAGCCTGCTGAAGCTCTTCATTCGGGAGTTGCC<br>CCAGCCACTGCTCAGTGTGGAGTATCTCAAAGCCTTTCAGGCTGTCCAGAATCTTCCAACCAAGAAGCAGCAA<br>CTACAGGCTTTGAACCTTCTTGTCATCCTCCTACCTGATGCAAACAGGGACACACTGAAGGCCCTTCTTGAAT<br>TTCTCCAAAGAGTAATAGATAATAAAGAAAAAAATAAAATGACAGTCATGAATGTAGCAATGGTCATGGCCCC<br>GAATCTCTTTATGTGTCATGCATTGGGATTGAAGTCCAGTGAACAGCGAGAATTTGTAATGGCAGCTGGGACA<br>GCAAATACCATGCACTTATTGATTAAGTACCAAAAACTTCTGTGGACAATTCCCAAGTTTATTGTAAACCAAG<br>TGAGGAAGCAAAACACGGAAAATCATAAAAAGGATAAAAGAGCCATGAAGAAATTGCTGAAGAAAATGGCTTA<br>TGACCGAGAAAAATATGAAAAGCAAGATAAGAGTACAAATGATGCTGACGTTCCTCAGGGAGTGATTCGAGTG<br>CAAGCTCCCCATCTTTCGAAAGTTTCAATGGCAATACAGCTAACTGAAGAACTAAAAGCCAGTGATGTACTTG<br>CCAGGTTTCTCAGCCAAGAAAGTGGGGTTGCCCAGACTCTCAAGAAAGGAGAAGTTTTTTTGTATGAAATTGG<br>AGGAAATATTGGGGAACGCTGCCTTGATGATGACACTTACATGAAGGATTTATATCAGCTTAACCCAAATGCT<br>GAGTGGGTTATAAAGTCAAAGCCATTGTAGAACTCGAGAATTCTGA
